# Direct calibration of PICKY-designed microarrays

**DOI:** 10.1186/1471-2105-10-347

**Published:** 2009-10-23

**Authors:** Hui-Hsien Chou, Arunee Trisiriroj, Sunyoung Park, Yue-Ie C Hsing, Pamela C Ronald, Patrick S Schnable

**Affiliations:** 1 Department of Genetics, Development and Cell Biology and Department of Computer Science, Iowa State University, Ames, IA 50011, USA; 2 Institute of Plant and Microbial Biology, Academia Sinica, Nankang, Taipei, 11529, Taiwan, Republic of China; 3 4000 S. Brahma Blvd. #E7, Kingsville, TX 78363, USA; 4 Department of Plant Pathology, University of California, Davis, CA 95616, USA; 5 Department of Agronomy, and Center for Plant Genomics, Plant Sciences Institute, Iowa State University, Ames, IA 50011, USA

## Abstract

**Background:**

Few microarrays have been quantitatively calibrated to identify optimal hybridization conditions because it is difficult to precisely determine the hybridization characteristics of a microarray using biologically variable cDNA samples.

**Results:**

Using synthesized samples with known concentrations of specific oligonucleotides, a series of microarray experiments was conducted to evaluate microarrays designed by PICKY, an oligo microarray design software tool, and to test a direct microarray calibration method based on the PICKY-predicted, thermodynamically closest nontarget information. The complete set of microarray experiment results is archived in the GEO database with series accession number GSE14717. Additional data files and Perl programs described in this paper can be obtained from the website  under the PICKY Download area.

**Conclusion:**

PICKY-designed microarray probes are highly reliable over a wide range of hybridization temperatures and sample concentrations. The microarray calibration method reported here allows researchers to experimentally optimize their hybridization conditions. Because this method is straightforward, uses existing microarrays and relatively inexpensive synthesized samples, it can be used by any lab that uses microarrays designed by PICKY. In addition, other microarrays can be reanalyzed by PICKY to obtain the thermodynamically closest nontarget information for calibration.

## Background

PICKY is an optimal oligo microarray design software developed for large and complex genomes [1]. PICKY-estimated DNA annealing temperatures for probes can deviate from actual annealing temperatures because some potentially important parameters are unavailable to its design algorithms, such as variations in the salt composition of hybridization buffers, effects of partially immobilized probes on the microarray surface, nonlinear and multistage nontarget annealing with a probe, effects of incorporated dye molecules on transcript annealing efficiency with a probe, and effects of additional chemicals (e.g., SDS, formamide or DMSO) in the hybridization buffers. These parameters vary with lab environments, and their influence on hybridization kinetics can only be experimentally measured. Because microarray experiments are complicated procedures involving many steps, multiple experiments must be conducted to provide statistically sufficient measurements. Objective microarray assessment can best be obtained from experiments that use controlled samples with precisely known compositions, i.e., by selecting a subset of probes and synthesizing their corresponding antisense oligonucleotides (oligos). If a subset is representative of the whole range of predicted probe annealing temperatures, it can calibrate most probe-target and probe-nontarget interactions.

For each probe it designs PICKY also predicts the thermodynamically closest nontarget transcript, i.e., the single transcript in the entire transcriptome other than the intended target transcript that is most likely to bind to a probe. These nontargets can be used to *calibrate *probe behaviors under a specific microarray protocol and various hybridization temperatures and sample concentrations. The calibration can then be used to determine the optimal hybridization conditions that can maximize the differentiation power of microarrays. The optimal conditions determined by this calibration can replace "rules of thumb" that are commonly being employed by the scientific community, e.g., by setting the array hybridization temperature such that 75~80% of probes "light up."

## Results

### Experiment design

The NSF 45K rice microarray [2,3] was chosen for this study because it was readily available to the authors and is representative of large-genome microarrays. It was designed on the basis of version 3 of the TIGR rice annotation, which contains 61 419 gene models [4]. PICKY 2.0 designed 43 311 probes on the microarray targeting 44 973 of the rice gene models; some probes target more than one gene.

**Figure 1:**
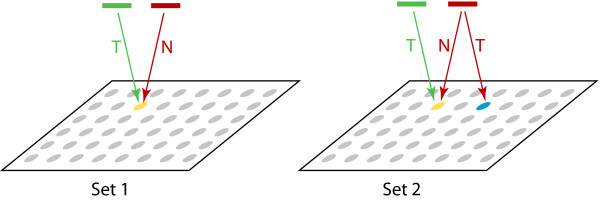
**Set 1 and Set 2 sample oligos**. Set 1 sample oligos are chosen as a pair for each probe on the microarray, where one is the probe target (green) and the other is its closest nontarget (red). Set 2 sample oligos are also chosen as a pair, but one is both the target and closest nontarget of two probes and can be used to establish a comparison basis between the two probes.

Two sets of synthesized samples were designed. Sample oligos in Set 1 were selected in pairs to test each probe on the microarray; one oligo in a pair is the intended target of a probe (green in Figure 1) and the other is the PICKY-predicted thermodynamically closest nontarget for the same probe based on an analysis of the remainder of the transcriptome (red in Figure 1). Sample oligos in Set 2 were also selected in pairs, but their selection criteria were more complicated. The nontarget antisense oligo in a pair also had to be the intended target of another probe (blue in Figure 1). On many PICKY-designed microarrays, transcripts targeted by some probes are also the closest nontargets to some other probes and have overlapping hybridization sites. Therefore, the antisense oligo synthesized to match the target region of a probe may by chance hybridize with another non-targeting probe as well. Set 1 is designed to measure probe competition between target and nontarget transcripts. Set 2 is designed to check transcript competition between two probes that can hybridize to the same transcript. The selection algorithms for Set 1 and Set 2 are detailed in the Methods section.

Pairs of sample oligos from both sets were synthesized, purified, diluted into different concentration levels, labeled with different Cy dyes and allowed to hybridize with probes on the microarray. The intensity ratios of the two colors on each probe were used to quantify how well the probes can distinguish their intended targets from their closest nontargets and to calibrate their optimal hybridization conditions. The goal of the calibration is to determine an optimal condition which allows both high target intensities (i.e., high sensitivity) and high ratios of target to nontarget intensities (i.e., high specificity). Identifying the thermodynamically closest nontarget transcript for each probe on a microarray is a unique feature of PICKY, so this experiment also examines whether these nontargets were recognized correctly.

Sample oligos were mixed and labeled with fluorescent dyes according to Table 1. Five experiments (E1~E5) were designed, each with different sample compositions. For example, in Set 1:E1, the probe 1 target oligo (*t1*) was labeled with Cy5 dye, and its nontarget oligo (**n1**) was labeled with Cy3 dye. Note that the concentration of *t1* is 10^4 ^times higher than that of **n1**, which represents the best-case scenario tested. Conversely, the probe 10 target (**t10**) concentration is 10^-4 ^weaker than its nontarget (*n10*), which represents the worst-case scenario tested. Set 2 target and nontarget oligos were always diluted to the same levels in each experiment; for example, in Set 2:E1, the probe 1 nontarget (**n1**) is set at the same level (10^-5^M) as the probe 1 target (*t1*).

**Table 1:** Oligo dilution levels and experiment design

**Set 1 sample oligos**					
**Conc.**	**E1**	**E2**	**E3**	**E4**	**E5**

**10^-5 ^M**	*t1***t6 n5***n10*	**t5***t7 n4***n6**	*t4***t8 n3***n7*	**t3***t9 n2***n8**	*t2***t10 n1***n9*
**10^-6 ^M**	*t2***t7 n4***n9*	**t1***t8 n3***n10**	*t5***t9 n2***n6*	**t4***t10 n1***n7**	*t3***t6 n5***n8*
**10^-7 ^M**	*t3***t8 n3***n8*	**t2***t9 n2***n9**	*t1***t10 n1***n10*	**t5***t6 n5***n6**	*t4***t7 n4***n7*
**10^-8 ^M**	*t4***t9 n2***n7*	**t3***t10 n1***n8**	*t2***t6 n5***n9*	**t1***t7 n4***n10**	*t5***t8 n3***n6*
**10^-9 ^M**	*t5***t10 n1***n6*	**t4***t6 n5***n7**	*t3***t7 n4***n8*	**t2***t8 n3***n9**	*t1***t9 n2***n10*

**Set 2 sample oligos**					

**Conc.**	**E1**	**E2**	**E3**	**E4**	**E5**

**10^-5 ^M**	*t1***t6 n1***n6*	**t5***t7 n5***n7**	*t4***t8 n4***n8*	**t3***t9 n3***n9**	*t2***t10 n2***n10*
**10^-6 ^M**	*t2***t7 n2***n7*	**t1***t8 n1***n8**	*t5***t9 n5***n9*	**t4***t10 n4***n10**	*t3***t6 n3***n6*
**10^-7 ^M**	*t3***t8 n3***n8*	**t2***t9 n2***n9**	*t1***t10 n1***n10*	**t5***t6 n5***n6**	*t4***t7 n4***n7*
**10^-8 ^M**	*t4***t9 n4***n9*	**t3***t10 n3***n10**	*t2***t6 n2***n6*	**t1***t7 n1***n7**	*t5***t8 n5***n8*
**10^-9 ^M**	*t5***t10 n5***n10*	**t4***t6 n4***n6**	*t3***t7 n3***n7*	**t2***t8 n2***n8**	*t1***t9 n1***n9*

*Italic underlined *letters = labeled with Cy5 dye. **Bold** letters = labeled with Cy3 dye.

Microarray hybridizations were conducted at 70°C, 60°C, 55°C, 50°C, 53°C, 45°C and 48°C according to the protocol summarized in the Methods section. The temperatures were adaptively selected by analyzing the results from prior hybridizations. At each temperature, 10 microarray slides were used to conduct experiments E1~E5 in duplicate. The 70°C results were discarded due to extremely weak signals. Each microarray slide was scanned multiple times at several PMT settings to maximize signal differentiation [5]. After removing some erroneous slides and duplicative scans, 165 valid GenePix Report (GPR) data files were produced. Note that Table 1 shows individual oligo dilution levels, but after pooling, drying and redissolving, the oligo concentrations during hybridizations were reduced to approximately 5 × 10^-8 ^M~5 × 10^-12 ^M.

### Data normalization

**Figure 2:**
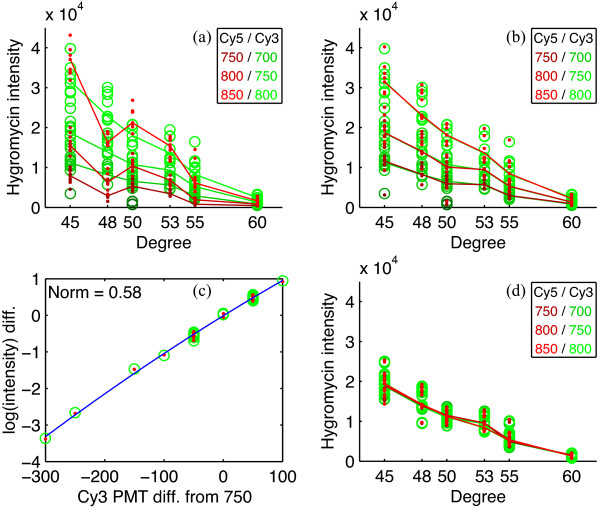
**Data normalization and scaling based on the hygromycin controls**. Measured dye intensities at different temperatures and under different PMT gains are shown. Lines connect the median intensities under the three most common pairs of Cy5/Cy3 PMT settings at each temperature. (a) Before normalization, the Cy5 (red) values are more variable. (b) After normalization, the Cy5 lines are aligned to Cy3 (green) lines, and most red dots are inside green circles. (c) Quadratic curve fit of the difference in log intensities against the difference in Cy3 PMT settings. (d) Intensity values can then be scaled toward the middle Cy3 750 line.

The NSF 45K array includes 457 hygromycin control spots that are distinctive to the wild-type rice genome. These spots are scattered among all 48 blocks on the array, thus providing a convenient means with which to normalize chips. Consequently, the target oligo for the hygromycin probe was also synthesized, diluted to 10^-7 ^M and added to each sample in either dye color to serve as a normalization control. After drying and redissolving, its final concentration was approximately 5 × 10^-10 ^M. Each of the 457 hygromycin spots was included in the normalization if it passed the quality criteria stated in the Methods. In Figure 2a, the average Cy5 (red) and Cy3 (green) intensities of the hygromycin spots are plotted versus hybridization temperature. The Cy5 channel is noticeably less monotonic with decreasing temperatures because it is more vulnerable to photo bleaching and ozone. Therefore, the Cy5 channel is normalized against the relatively more stable Cy3 channel. Figure 2b shows normalized hygromycin intensities. The two intensities of each array were averaged from all hygromycin spots, but the normalization factor is determined from the log average of individual two-color regression ratios; thus, they are expected to be close but not necessarily the same. All other probe intensities were normalized accordingly. The background-subtracted and normalized median intensities were used in subsequent analyses. Calibration without background subtraction also works but is less precise because autofluorescence background can sometimes overwhelm weak nontarget signals (data not shown). If an intensity value was less than zero, it was reset to zero. All intensity values were then increased by one to avoid division by zero or a negative infinite logarithm.

Scanning microarrays at different PMT settings produces several different sets of intensity values from the same microarray. Although this scanning improves estimates of the ratio between the two channels for each probe, it introduces an artificial variability into the intensity values. For some of our analyses that use absolute intensity values, it is preferable to remove this variability. A regression on the difference of the log of the average hygromycin intensities is performed against the difference in PMT settings to scale the intensities from the same microarray all toward the middle Cy3 750 line. Because the Cy5 values have already been normalized against Cy3 values, they are scaled similarly. The regression is shown in Figure 2c; the PMT-versus-intensity relationship is almost linear, but a quadratic fit provides a slightly better norm of residues (0.71 vs. 0.58). Figure 2d shows the final scaled intensity values.

## Discussion

### Set 1 data analysis

**Figure 3:**
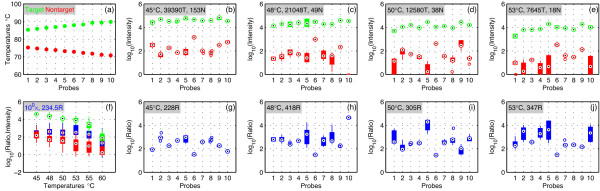
**Set 1 results under equal target and nontarget concentrations**. (a) PICKY-predicted target (green) and nontarget (red) annealing temperatures for the chosen probes. (b-e) Measured **T**arget and **N**ontarget intensities of individual probes at different temperatures; the median values among probes at each temperature are shown in the label. (f) Combined target intensities (green), nontarget intensities (red) and their ratios (blue) across all hybridization temperatures. (g-j) Target and nontarget intensity **R**atios of individual probes at different temperatures; the median values among probes are shown in the labels. In these figures, boxes enclose measured values within the lower and upper quartiles, circled dots indicate median values, whisker lines extending from either end of the boxes show the extent of the rest of data, and isolated empty circles indicate outliers.

The PICKY-computed annealing temperatures between Set 1 probes and their targets and closest nontargets are shown in Figure 3a. As mentioned earlier, these calibration probes were selected such that they span the entire range of annealing temperatures for the 43 111 probes on the rice microarray. The target annealing temperatures were directly calculated by using the nearest-neighbor (N-N) model [6]; nontarget annealing temperatures were estimated by the method described [1]. The first task of our data analysis was to compare how the measured probe behaviors reflect these computed characteristics.

There are several variables, including hybridization temperatures, specific probes and the ratios of target to nontarget concentrations. To start easy, consider only equal concentrations of targets and nontargets, i.e., only results produced from the third row of Table 1. After normalization and scaling, the dye intensities of Set 1 probes at different hybridization temperatures are shown in Figures 3b-e. The green and red colors indicate the target and nontarget intensities, not the actual dyes used to label samples. For the targets, as expected, higher intensities are measured at lower hybridization temperatures. Among probes, the relative target intensities also follow the predicted trend in Figure 3a, i.e., probes with higher annealing temperatures produce stronger intensities because they bind stronger to their targets at the same hybridization temperature. A similar trend is observed for the nontargets as well, although they are more variable because of their generally weaker signals than targets. This observation provides evidence that PICKY's closest nontarget predictions based on thermodynamics are sensible.

The ratios of target to nontarget hybridization intensities are shown in Figures 3g-j. For each probe, the ratios are more variable at higher temperatures but become stable when the temperature is lowered (i.e., the boxes representing the distribution of ratios at each probe are largely reduced to single dots at 45°C). Although the variability in per-probe ratios is lowest at 45°C, the combined median ratio among probes drops sharply to 228. At the slightly higher 48°C, the highest median ratio 418 can be achieved with slightly higher variability. In Figure 3f, the individual probe intensities and ratios are statistically combined and plotted against the hybridization temperature. This figure indicates that PICKY-designed probes can maintain hundreds of times stronger target-to-nontarget intensities over a wide temperature range (45~55°C), at least when targets and nontargets are present at equal concentrations (~5 × 10^-10 ^M).

**Figure 4:**
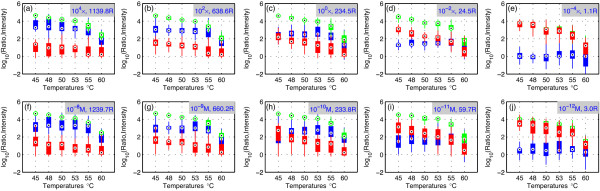
**Set 1 and Set 2 results under all target and nontarget concentrations**. The measured target intensities (green) and nontarget intensities (red) and their ratios (blue) at different (a-e) Set 1 target-to-nontarget concentration ratios and (f-j) Set 2 target and nontarget absolute concentrations are drawn against the hybridization temperatures. Also labeled in each figure is the median ratio value for the presented subset of data in that figure. In these figures, boxes enclose values within the lower and upper quartiles, circled dots indicate median values, and whisker lines extending from the ends of boxes show the extent of the rest of data. To avoid clutter, outliers are not plotted. In these figures temperatures are categorical data and are therefore not plotted to scale on the x-axis.

Figures 4a-e now expands the comparisons to include all Set 1 data, with sample target and nontarget concentration ratios ranging from 10^4 ^to 10^-4^. For favorable ratios ≥10^0 ^= 1, PICKY-designed probes always exhibit target intensities that are hundreds of times stronger than closest nontarget intensities. At the less favorable ratio of 10^-2^, where targets are 100 times more diluted than nontargets, probes can still differentiate targets from nontargets, but their resolution power (i.e., ratios) are influenced by the hybridization temperature. This finding demonstrates that the common strategy of simply lowering hybridization temperatures to boost signal intensities reduces specificity and hence the quality of the microarray data [1,7]. Only when the targets are 10^-4 ^more diluted than the nontargets will the probes become unable to distinguish targets from nontargets, i.e., to exhibit ratios of target to nontarget intensities close to 1. In practice, this observation means that detections could be missed (false negatives) but there are probably no incorrect detections (false positives).

### Set 2 data analysis

Set 2 targets and nontargets are always at the same absolute concentration levels, as labeled in Figures 4f-j. In each vertical pair of Set 1 and Set 2 figures, the target intensities are largely equal because they are diluted to the same level (e.g., Figures 4a and 4f represent targets at the same 10^-8 ^M concentration level). The most surprising results come from Set 2 nontargets, whose intensities seem to be steadily *increasing *with *decreasing *sample concentrations (e.g., compare nontarget results shown in red for a given temperature across Figures 4f-j). Unlike Set 1 nontargets, which are at opposite levels of concentration than the targets (cf. Table 1), such behaviors are not expected for Set 2. The dedicated probes for Set 2 nontargets (cf. Figure 1) confirm that Set 2 nontarget concentrations are indeed reduced (data not shown). We hypothesize that nontarget binding to probes is strongly attenuated by target concentration. When perfect targets are abundant, they compete with nontargets. This finding explains why Set 2 nontarget intensities are not much stronger than Set 1 nontarget intensities even though Set 2 nontargets are at 10^4 ^× higher concentration (compare Figures 4a↔f). At lower target concentrations, more probe molecules might be available to bind to nontargets. Hence, even though the nontargets are present at reduced concentrations; their signal intensities can actually increase, as observed in Figures 4f-j, due to reduced competition from targets.

Set 2 was designed to test transcript competition, i.e., the potential dilution effect of two probes both binding to the same transcript. This type of effect was not seen in our results. Therefore, probe competition seems to be the main determinant of target and nontarget binding strength. Our hypothesis agrees with similar explanations independently offered by others [8,9]. Nevertheless, probe competition is not formulated into existing microarray design algorithms (e.g., PICKY estimates the melting temperature between any pair of probes and transcripts as if they were the only two participants in a microarray reaction). At the moment, it is not clear how to reliably estimate the complex kinetics of many competing transcripts binding with many probes at the same time. Although a coupled kinetics analysis has recently been developed to model two transcripts binding to two probes [10], scaling up the analysis for general microarray design involving tens of thousands of probes and transcripts is difficult. Despite this design limitation, the characteristics of microarray probes can be empirically calibrated to determine the optimal hybridization conditions of a microarray according to the data collected from Set 1 and Set 2 probes.

### Microarray calibration

**Figure 5:**
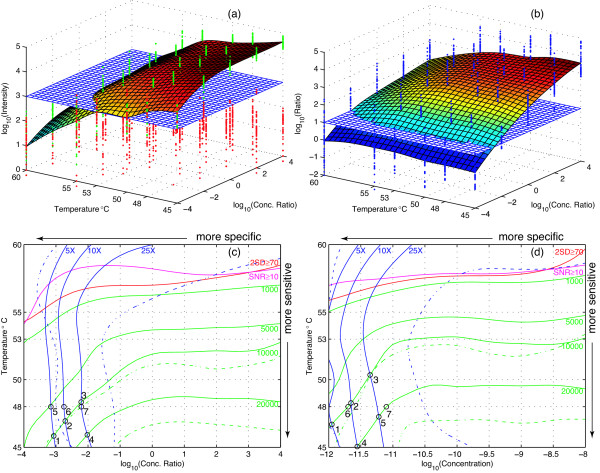
**Microarray calibration**. (a) Set 1 target intensity as a calibrated function of sample concentration ratio and hybridization temperature. Green and red dots represent the measured target and nontarget values. (b) Set 1 intensity ratio similarly calibrated as a function of sample concentration ratio and hybridization temperature; blue dots are the measured ratio values. (c) Contour map of several intensity (green), ratio (blue), SNR (magenta) and 2SD (red) lines obtained from Set 1 data. (d) Contour map of similar set of lines obtained from Set 2 data. Dashed lines in the contour maps enclose the ± 1 standard deviation range around the 20000 intensity line and the 25× ratio line. See Table 2 for the numerical coordinates of the intersecting points 1--7 on the two contour maps.

Shown in Figure 5a is the Set 1 target intensity surface calibrated using signal strengths for the hybridization of targets at various sample concentration ratios and hybridization temperatures (shown as green data points). The red data points represent signal strengths for the hybridization of nontargets. Blue target-to-nontarget intensity ratio points shown in Figure 5b are similarly used to calibrate the ratio surface. The two surfaces have very different inclinations. Target intensities are more dependent on hybridization temperature, whereas intensity ratios are more sensitive to sample concentration ratios. To compare the two surfaces, contour lines representing fixed levels on each surface can be projected onto the same 2D space. For example, in Figure 5a, the target intensity surface intersects the log_10_(1000) = 3 level plane and forms the green 1000 contour line in Figure 5c. Similarly, in Figure 5b, the ratio surface intersects the log_10_(10) = 1 level plane and forms the blue 10× contour line in Figure 5c. The 10000 and 20000 intensity lines cross the three ratio lines at points 1-4 listed in Table 2c. In addition, two quality control lines are drawn: the magenta line marks where target signal-to-noise ratios (SNRs) rise above 10, and the red line marks where pixels two standard deviations above the background exceed 70%. These two quality lines are also calibrated from the data. Together with the 1000 intensity line, these are the three most commonly used quality controls for microarrays. Indeed they are very consistent with each other and carve out a top region on the contour map that should be avoided. Figure 5d shows a similar set of contour lines based on Set 2 data. Of note, its three ratio lines are shifted toward the left, which indicates that Set 2 probes are more specific at lower sample concentrations. This result is expected because nontargets in Set 1 are more concentrated at lower target concentrations and may interfere in target bindings with probes, as was previously hypothesized.

**Table 2:** Contour line intersecting points in Figure 5

**(c) Set 1 intersection points**				
**Point**	**Conc. ratio**	**Temp.**	**Intensity**	**Ratio**

**1**	-3.07	45.84	10000	5
**2**	-2.71	46.95	10000	10
**3**	-2.21	48.34	10000	25
**4**	-2.02	45.93	20000	25
**5**	-3.16	48.00	5217	5
**6**	-2.75	48.00	7270	10
**7**	-2.21	48.00	11167	25

**(d) Set 2 intersection points**				

**Point**	**Conc. (M)**	**Temp.**	**Intensity**	**Ratio**

**1**	-11.95	46.69	10000	5
**2**	-11.65	48.27	10000	10
**3**	-11.36	50.36	10000	25
**4**	-11.55	45.05	20000	10
**5**	-11.22	47.26	20000	25
**6**	-11.70	48.00	10000	9
**7**	-11.10	48.00	20000	35

Conc. ratio or Conc. (M) values are in log_10 _scale.

### Optimal hybridization conditions

The most important condition for microarrays that researchers need to determine is the hybridization temperature. With different microarrays and under different lab protocols, the optimal choice often varies. There are conflicting concerns when choosing the optimal hybridization temperature. As seen from the two contour maps in Figure 5, higher microarray sensitivity (i.e., higher measured intensities) can be achieved when the hybridization temperature is lowered, but this conflicts with the goal to also achieve higher specificity (i.e., higher target-to-nontarget intensity ratios) because the ratio lines all gradually shift toward the right after peaking at 53~55°C. Setting hybridization at such high temperatures, however, will reduce target intensities below the 5000 level, even for the most concentrated samples.

Because microarray experiments are inherently noisy, these contour lines should be interpreted as median division lines with half of the expected data on either side of a line. For example, on the two contour maps, the dashed green lines indicate the calibrated ± 1 standard deviation boundaries around the 20000 intensity lines and the dashed blue lines similarly enclose the 25× ratio lines. Our results suggest a normal distribution of intensity values on the log scale with the measured average standard deviation of 0.56. Therefore, at medium intensity of log_10_(5000), about 10.5% of target intensities are expected to fall below the minimum 1000 level. If the medium intensity is at log_10_(10000), then 3.7% of the target intensities may fall below 1000. At log_10_(20000), less than 1% may do so. Although the actual standard deviation also depends on temperature and sample concentration, this estimate suggests that the median intensity line should be controlled to be no less than 10000.

The minimal ratio line should be decided by microarray users according to their domain knowledge and specific application. For example, if samples are directly converted from mRNA without amplification, then probe sensitivity may be more important to the users. In this case, users may choose the 5× ratio line and follow it to point 1 in Figure 5d where it intersects the 10000 intensity line. This tells them to set their hybridization temperature at 46.7°C. In contrast, if a genome under study contains many paralogous genes and users are concerned about the specificity of low-copy transcript detection, they may wish to follow the 25× ratio line to point 3 in Figure 5c and set their hybridization temperature at 48.3°C. Both contour maps may be used to determine the optimal hybridization conditions specific to a user. Figure 5c is based on Set 1 data and may be considered the worst-case scenario; Figure 5d is based on Set 2 data and may be considered a more typical scenario. Without special concerns of the samples, it is recommended to set the hybridization temperature to the intersection point of the 10000 intensity line and 10× ratio line on Figure 5d, which is 48°C for the NSF rice microarray as previously recommended.

### Numerical predictions

**Figure 6:**
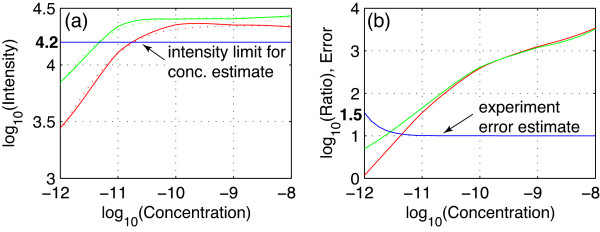
**Predictions of probe behaviors at 48°C hybridization temperature**. Set 1 (red lines) and Set 2 (green lines) calibration results as functions of target sample concentration. (a) Calibrated target intensities (solid lines) and their regression equations (dotted lines) at 48°C. The horizontal blue line limits intensity-to-concentration reverse estimates to the linear range. (b) Target-to-nontarget intensity ratios lines. The blue line represents estimated experiment errors attributable to nontarget background differences; note that it is in linear scale.

Once the hybridization conditions have been decided, some predictions can also be made on the basis of the calibration data. For example, the NSF rice microarray team previously recommended 48°C for hybridization. This temperature intersects ratio lines at points 5-7 in Figure 5c and intensity lines at points 6 and 7 in Figure 5d. In fact, the intersecting lines at 48°C can be directly obtained from the intensity and ratio surfaces of Set 1 and Set 2 data. These lines express intensity and ratio as functions of target concentration as shown in Figure 6. Assuming that measured dye intensities are linearly correlated to bound DNA on the probes, the intensity function can be approximated by the Langmuir kinetic model for adsorption [11]. This model produces the following regression equations based on the data presented in Figure 6a, where *I *is the intensity and *C *is the target concentration:

For Set 1: *I *= 22185 × *C*/(7.57 × 10^-12 ^+ *C*)

For Set 2: *I *= 26272 × *C*/(2.94 × 10^-12 ^+ *C*)

These equations describe the intensity curves well (see the dotted lines in Figure 6a) and can be reversed to estimate sample concentrations at some measured intensity values within the linear range (i.e., when *I *≤ 10^4.2^). For example, at the intensity of 5000, Eq. 1 predicts 10^-11.7 ^M and Eq. 2 predicts 10^-12.2 ^M target concentrations. According to Figure 6a prediction, to keep intensities above 10000, users should maintain sample concentrations above 10^-11.2 ^M.

Despite their different sample compositions, Set 1 and Set 2 target-to-nontarget intensity ratios in Figure 6b do not show visible differences above the 10^-10.5 ^M target concentration. Here, **r**atios are between **t**arget and **n**ontarget intensities, i.e., *r *= *t*/*n*. In regular microarray applications, both intensities are in the same color for each sample and the normal fold change ratios are determined as *R *= (*t*_1_+n_1_)/(*t*_2_+n_2_), where subscripts 1 and 2 denote the two different samples being compared. This equation can be factorized as *R *= (*t*_1_(1+ 1/*r*_1_))/(*t*_2_(1+1/*r*_2_)). If the target concentration does not change (i.e., *t*_1 _= *t*_2)_, then *E *= (1+ 1/*r*_1_)/(1+1/*r*_2_) can be used to estimate the error caused by the different nontarget background between the two samples. Assuming one sample has an opposing nontarget concentration similar to Set 1 data and the other sample has roughly the same target and nontarget concentration similar to Set 2 data, the error can be estimated by the two ratio lines in Figure 6b and is drawn in linear scale as the blue line. This blue line predicts that for the PICKY-designed rice 45K microarray, no noticeable ratio error caused by different nontarget backgrounds is expected when the target concentration is above 10^-11.2 ^M. The estimated maximum ratio error is 1.54 at the lowest target concentration, but most statistical analyses will not consider ratios lower than 2 to be significant; thus, this error is unlikely to cause false discovery.

## Conclusion

This study uses PICKY-designed probes that have already been optimized; it is not possible to know how microarray quality might degrade if certain PICKY design criteria were removed, or which criteria are more important than others. A recent large-scale study involving millions of oligonucleotide probes to evaluate the probe quality associated with various probe design criteria highlights the most influential factor of microarray signal intensity: the target melting temperature [8]. Many microarray design tools choose probes with higher melting temperatures because they produce stronger hybridization intensities. This study shows that probes with higher melting temperatures also produce more nonspecific binding. The PICKY design strategy is not biased toward high melting temperatures. Instead, it optimizes the *uniformity *and the *separation *of target and nontarget melting temperatures among all probes [1]. Therefore, the calibration method presented here is likely to improve the overall quality of PICKY-designed microarrays despite the fact that only a small subset of probes is being calibrated. Furthermore, the same study suggested that variable-length probes be designed to improve the overall thermal uniformity among all microarray probes, which is also a key feature in PICKY.

The results from this work show that PICKY-designed microarray probes are robust and consistent throughout a wide range of temperature and concentration. Recent biological studies also demonstrate their high quality [2,12]. PICKY's prediction of the thermodynamically closest nontarget transcript of each probe is used to calibrate the microarray. Although not all nontargets of each probe are considered, chances are low for PICKY-designed probes to have many equally strong nontargets; such probes would not have been chosen by PICKY. Therefore, it is only necessary to calibrate the behavior of the closest nontargets for each probe. If we can prevent them from binding to the probes, all other less-potent nontargets are under control as well. This method does not require any special instrument or skill other than the small set of synthesized sample oligos used for calibration. Therefore, it can be readily applied by microarray users to improve their experimental results. Microarrays not designed by PICKY can be analyzed via PICKY's microarray reanalysis function to obtain the closest nontarget information. Therefore, the calibration method described in this study could in principle be used to calibrate any microarray. Nevertheless, if the probes on such microarrays were not uniformly designed, more calibration probes may be needed to improve the precision of calibration than are necessary to calibrate microarrays designed by PICKY.

## Methods

### Set 1 and Set 2 sample selections

The following screening steps were conducted to select a limited set of calibration probes:

(1) Ignore probes whose closest nontargets are not in the same read direction as the input gene models.

(2) Ignore probes whose closest nontargets do not have their own probes on the microarray.

(3) Record the sequence of each targeted gene model to be able to identify antisense sample oligos with dangling ends.

(4) Sort the remaining probes according to their PICKY-estimated target and nontarget melting temperature differences.

(5) Perform the following selection steps until enough (e.g., 1000) calibration probe candidates are found:

1. Align probes with their target genes and extract the target antisense oligo with two extra dangling end DNA bases.

2. Align probes with their nontarget genes and skip those whose aligned region is not ± 15 bp centered on the probes.

3. For Set 1, skip probes whose aligned regions on nontargets overlap the nontargets' own probe target regions. For Set 2, the non-overlapping probes are skipped.

4. The nontarget sample oligos for Set 1 are then chosen to match the probe, and the nontarget sample oligos for Set 2 are chosen to match their own probes.

Steps 1 and 2 are conducted to avoid expressed antisense transcript interference and to allow nontarget expression confirmation based on their own probes if natural samples are used for the calibration; they are not strictly necessary for our experiments that use synthesized samples. Although the dangling end bases are considered during PICKY calculation [13], they cannot be determined from the PICKY output file and must be read back from the original gene sequences in step 3. For Set 1, we try to avoid the potential dilution effect where the nontarget oligos can also anneal to their own probes. For Set 2, this is by design. Therefore, there are two different selection criteria in step 5.3. Two Perl programs *sel1.pl *and *sel2.pl *are written to perform the selections, and two more Perl programs *choose1.pl *and *choose2.pl *can be applied on the selected sets to choose sample oligo pairs that evenly span the range of melting temperatures and provide a representative coverage of the whole microarray. A final manual reduction step limiting each set to 10 sample oligo pairs for synthesis has been conducted due to cost concerns. The chosen probes and oligos for the experiments are outlined in the supplementary file "set1_and_set2_chosen.xls".

### Oligo synthesis and sample preparation

Sample oligos were synthesized by Integrated DNA Technologies [14] using their ion-exchange HPLC method to ensure full-length products. The oligos were resuspended in water and diluted to the calculated concentrations of 10^-5^~10^-9 ^M. The measured average, median and standard deviation concentrations for the 10^-5 ^M level oligos were 1.62 × 10^-5 ^M, 1.57 × 10^-5 ^M and 0.27 × 10^-5 ^M, respectively. It is not possible to accurately measure concentrations below the 10^-7 ^M level (~1 ng/μl) due to the sensitivity limits of our Nanodrop spectrophotometer. All measured concentrations are documented in the supplementary file "Measured DNA Concentrations.xls". For each experiment E1~E5, 10 μl of the 10 oligos in the same color from each set were pooled into the same tube. The antisense oligo for the hygromycin probe was also added (1 μl) to each tube, and 10 μl of the tube content were then removed to be coupled with Cy3 or Cy5 fluorescent dyes. All synthesized oligos include a 5' amino modifier C6 for direct dye incorporation. Uncoupled dyes were removed from labeled oligos using the QIAquick PCR Purification Kit (Qiagen). Final samples were pooled and dried in a vacuum dryer at 55°C.

### Microarray hybridization and image analysis

Microarray hybridizations were conducted as described [15]. A 200-μl hybridization buffer (made from 60 μl formamide, 50 μl of 20× SSC buffer, 10 μl of 2% SDS, 10 μl of 1 μg/μl hCOT I DNA, 10 μl of 1 μg/μl poly A and 1 μl of 20 μg/μl yeast tRNA and filled with water to 200 μl) was used to dissolve labeled samples. The buffer was heated at 95°C for 30 sec, and applied to the microarray. Microarray slides were pre-hybridized for 30 min with the pre-hybridization buffer (500 μl of 10 mg/ml bovine serum albumin, 12.5 ml of 20× SSC buffer and 250 μl of 20% SDS filled with water to 50 ml). Hybridizations were conducted using the automatic Lucidea SlidePro Hybridizer (Amersham Biosciences) for 16 hr at specified hybridization temperature.

Microarray slides were scanned using a GenePix 4100A scanner and analyzed using GenePix Pro 6 analysis software (Molecular Devices). The GenePix Array List "rice_45k.v4.feb1706.gal" for the 45K rice array was provided by TIGR and used to recreate the 48 array grids in GenePix (supplementary document). The grids were first automatically aligned to the array images and then manually adjusted grid by grid to match the spots. Analysis results were saved into GenePix Report (GPR) files. Each hygromycin control spot was included in the normalization if it passed the following quality criteria: no GenePix error flag raised; regression Cy5/Cy3 ratio is 0.1~10; both colors have ≥70% pixels two standard deviations above the background; the background-subtracted median intensity ratio is within 80%~120% of the regression ratio; and the regression coefficient of determination is >0.5. All other probe intensities were included if no GenePix error flag had been raised.

## Availability

The complete set of programs, code libraries and sample data files to illustrate how to select calibration probes based on the PICKY design output and how to harvest the calibration results for analysis can be downloaded from the PICKY website [16]. These files provide both a tutorial of the calibration procedure and sample code that can be modified to work on other data files. See the included README.txt for more details of each supplementary file. The complete set of microarray experiment results is archived in the GEO database with series accession number GSE14717 [17].

## Authors' contributions

HC developed the PICKY software, conceived of the calibration method, analyzed the generated data, and drafted the manuscript. AT performed sample preparations and microarray experiments. SP quantified microarray images to obtain raw intensity values. YCH participated in the experiment design and revised the manuscript. PCR and PSS created the rice microarrays used for this study, participated in the experiment design, and revised the manuscript. All authors read and approved the final manuscript.
